# miR-217 inhibits laryngeal cancer metastasis by repressing AEG-1 and PD-L1 expression

**DOI:** 10.18632/oncotarget.19121

**Published:** 2017-07-10

**Authors:** Susheng Miao, Xionghui Mao, Shu Zhao, Kaibin Song, Cheng Xiang, Yuanjing Lv, Huanyv Jiang, Lei Wang, Baojun Li, Xianguang Yang, Zhennan Yuan, Cheng Xiu, Hongxue Meng, Ji Sun

**Affiliations:** ^1^ Department of Head and Neck Surgery, Harbin Medical University Cancer Hospital, Harbin 150081, China; ^2^ Department of Pathology, Harbin Medical University Cancer Hospital, Harbin 150081, China; ^3^ Department of Medical Oncology, Harbin Medical University Cancer Hospital, Harbin 150081, China

**Keywords:** laryngeal cancer, metastasis, mir-217, AEG-1, PD-L1

## Abstract

High incidences of laryngeal cancer have been reported recently. Increasing our understanding of the molecular mechanisms underlying this malignancy could reveal more effective approaches to treating laryngeal cancer patients and so improve their prognoses. In this study, we explored the biological effects of miR-217 on laryngeal cancer. miR-217 potently inhibited multiple metastatic traits, including cell migration, invasion, proliferation, apoptosis, and EMT, as well as angiogensis. These effects were achieved through downregulation of the miR-217 target gene, AEG-1 and PD-L1. Clinical expression and animal model studies further confirmed our results. These findings provide new insight into the physiological effects of miR-217 in laryngeal cancer and its potential therapeutic use.

## INTRODUCTION

Laryngeal cancer is the most common head and neck cancer in the world, and its incidence has been recently increasing [1]. The main treatment strategy for laryngeal cancer is surgery or total laryngectomy. Therefore, there is an urgent need for understanding the mechanisms underlying laryngeal cancer pathogenesis. Metastasis is the overwhelming cause of mortality in patients with solid tumors [2], and has therefore received much attention in research studies [3]. Nonetheless, our knowledge of the molecular mechanisms underlying metastasis in laryngeal cancer is still scarce [4, 5].

As a new class of signaling modulators, microRNAs (miRNAs) negatively regulate gene expression by binding to the 3’UTR of target mRNAs, in a post-transcriptional manner [6]. A small number of miRNAs have been demonstrated to actively promote or inhibit carcinogenesis and metastasis [7–11]. Furthermore, miRNAs cab also promote the initiation and progression of most types of cancers [11]. One such miRNA is miR-217, which promotes several types of cancers, including esophageal and ovarian cancers, as well as glioma [12–14]. However, the clinical effects of miR-217 inlaryngeal cancer have not been fully elucidated.

In this study, we explored the role of miR-217 in the development of laryngeal cancer. According to our results, overexpression of miR-217 in laryngeal cancer Hep2 cells resulted in inhibition of cell migration, invasion and proliferation, together with an increase in cell apoptosis and induction of G1 phase arrest. We also showed that overexpression of miR-217 inhibited EMT and angiogenesis. Furthermore, we identified AEG-1 and PD-L1 as direct targets of miR-217 in laryngeal cancer. Of note, AEG-1 activated PD-L1 at the transcriptional level. Lastly, our clinical evidence and animal model studies substantiated our results. Therefore, our work has uncovered potential clinical targets for the treatment of laryngeal cancer.

## RESULTS

### Decreased expression of miR-217 in human laryngeal cancer tissues

**Figure 1:**
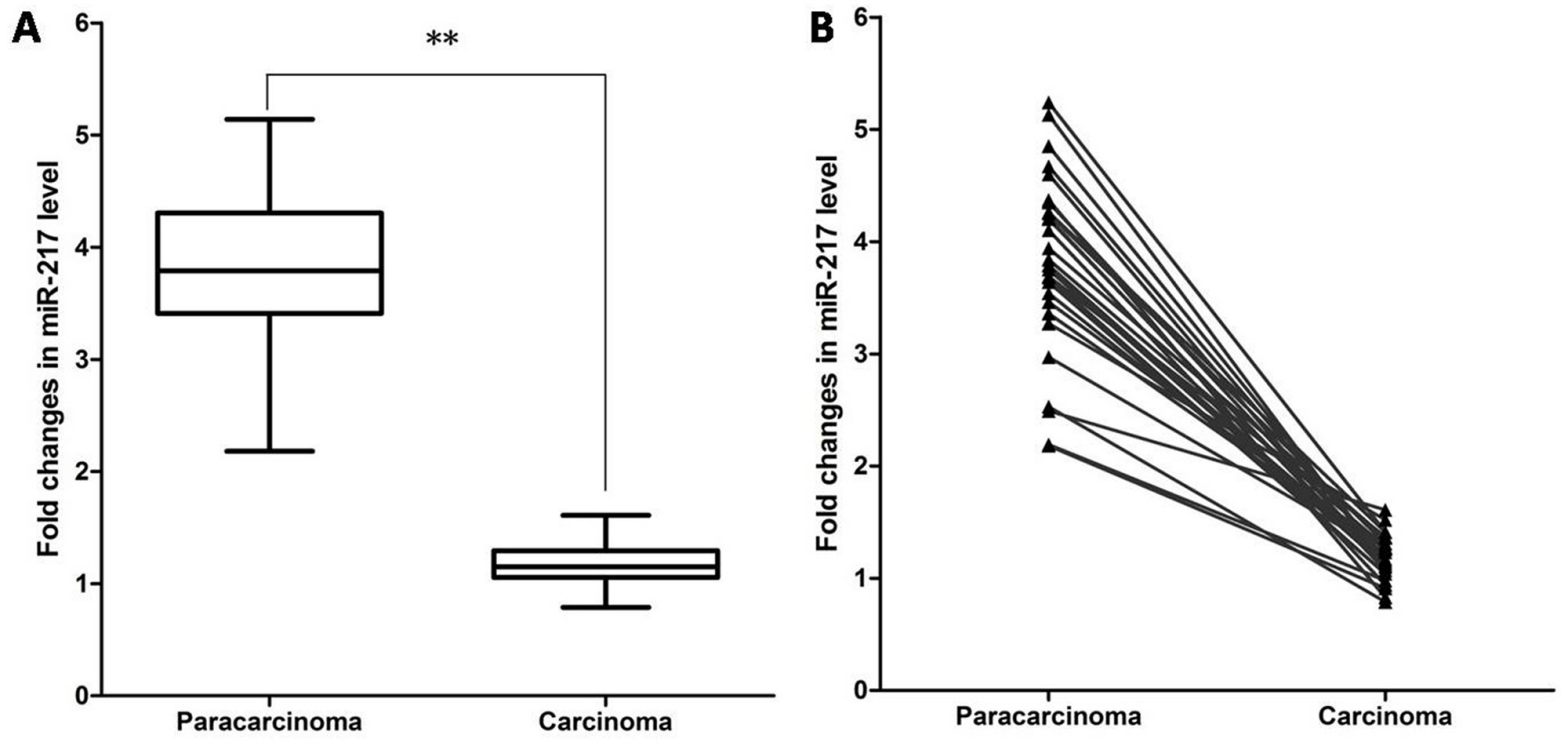
Expression of miR-217 in laryngeal cancer tissues miR-217 levels were determined by qRT-PCR. **(A)** Box-plot of miR-217 expression patterns in carcinoma and paracarcinoma tissues (N=29, P < 0.0001). **(B)** Pairwise comparison of miR-217 expression between carcinoma and matching paracarcinoma tissues showing that miR-217 expression was reduced in 29/29 of the sample pairs.

To explore if miR-217 might have a role in carcinogenesis of human laryngeal cancer tissues, qRT-PCR was first applied to determine its expression level in carcinoma tissues and adjacent paracarcinoma tissues, and normalized to U6 small RNA expression. The results showed that miR-217 levels in laryngeal carcinoma tissues were significantly decreased compared to those in paired normal tissues (29 pairs, P < 0.0001) **(**Figure 1A). Pair-wise comparisons indicated that more than 90% of tumors showed greater than 2-fold reduction of miR-217 expression compared to their matching controls (Figure 1B).

### miR-217 inhibits metastatic traits in laryngeal cancer cells

**Figure 2:**
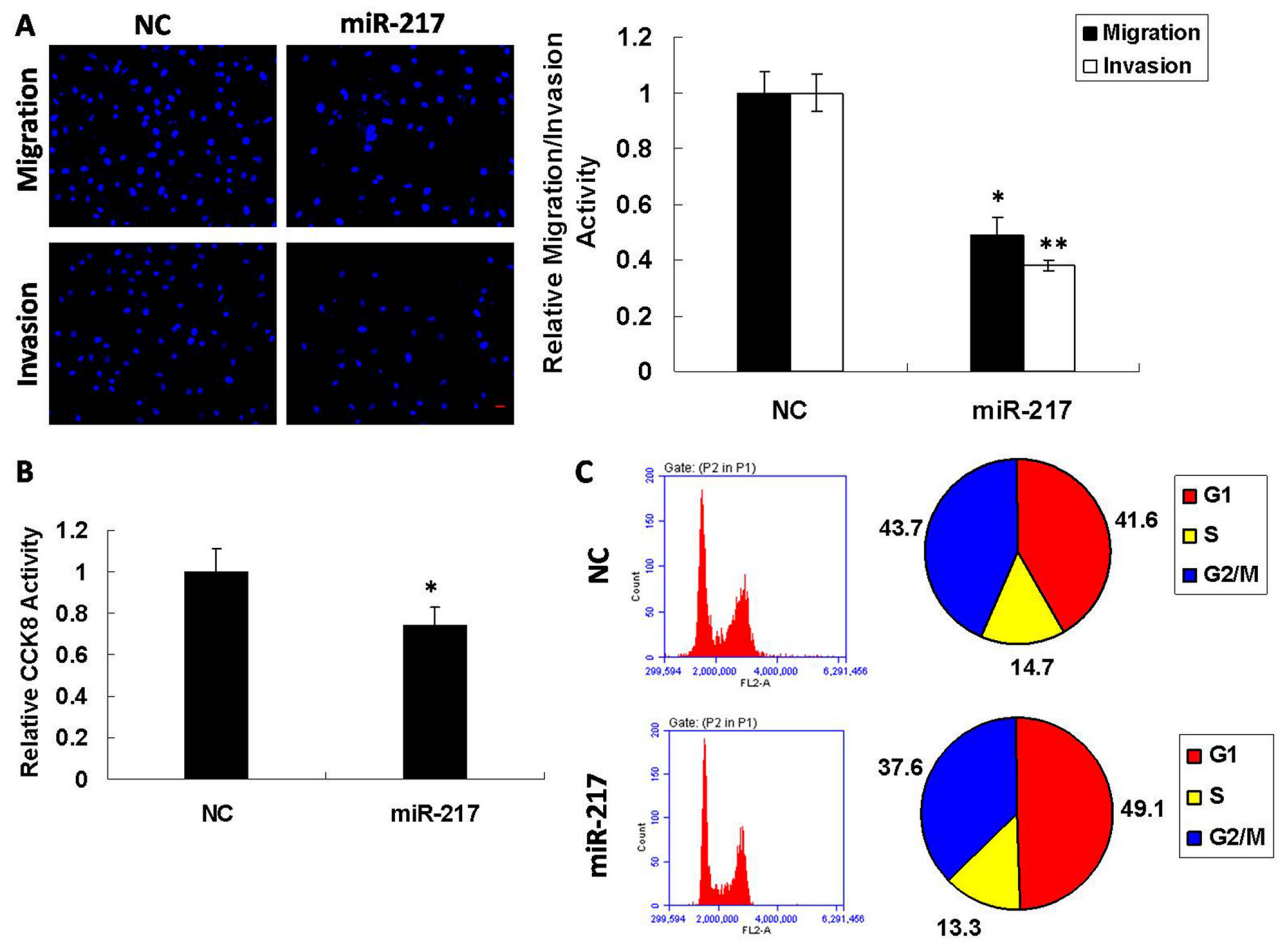
miR-217 modulates metastatic traits in Hep2 cells Overexpression of miR-217 in Hep2 cells resulted in **(A)** reduced migratory and invasive capability (N=5; scale bar, 50 μm), **(B)** reduced cell viability (N=3), and **(C)** cell cycle arrest (N=3).

To explore the possible role of miR-217 in laryngeal cancer cells, we conducted functional experiments using Hep2 cell lines. When miR-217 mimics and NC (negative control) were transfected into Hep2 cells individually by transwell migration and invasion assay, the migratory capabilities of the cells with high expression of miR-217 showed a 50% reduction compared to those transfected with NC, and invasion was reduced to 40% (Figure 2A). We additionally measured cell proliferation by CCK8 assay and found that overexpression of miR-217 moderately inhibited cell proliferation (Figure 2B). Quantitative cell cycle FACS analysis revealed that miR-217 increased the proportion of cells in G1 phase with a concomitant decrease in G2/M phase compared to control (Figure 2C). Furthermore, miR-217 overexpression in Hep2 cells promoted both apoptosis and necrosis compared to the control group via both Annexin V/PI staining assay (Supplementary Figure 1) and Caspase-3 activity assay (Supplementary Figure 2). These findings showed that miR-217 inhibited metastatic traits in laryngeal cancer cells.

### miR-217 inhibits EMT in laryngeal cancer cells

**Figure 3:**
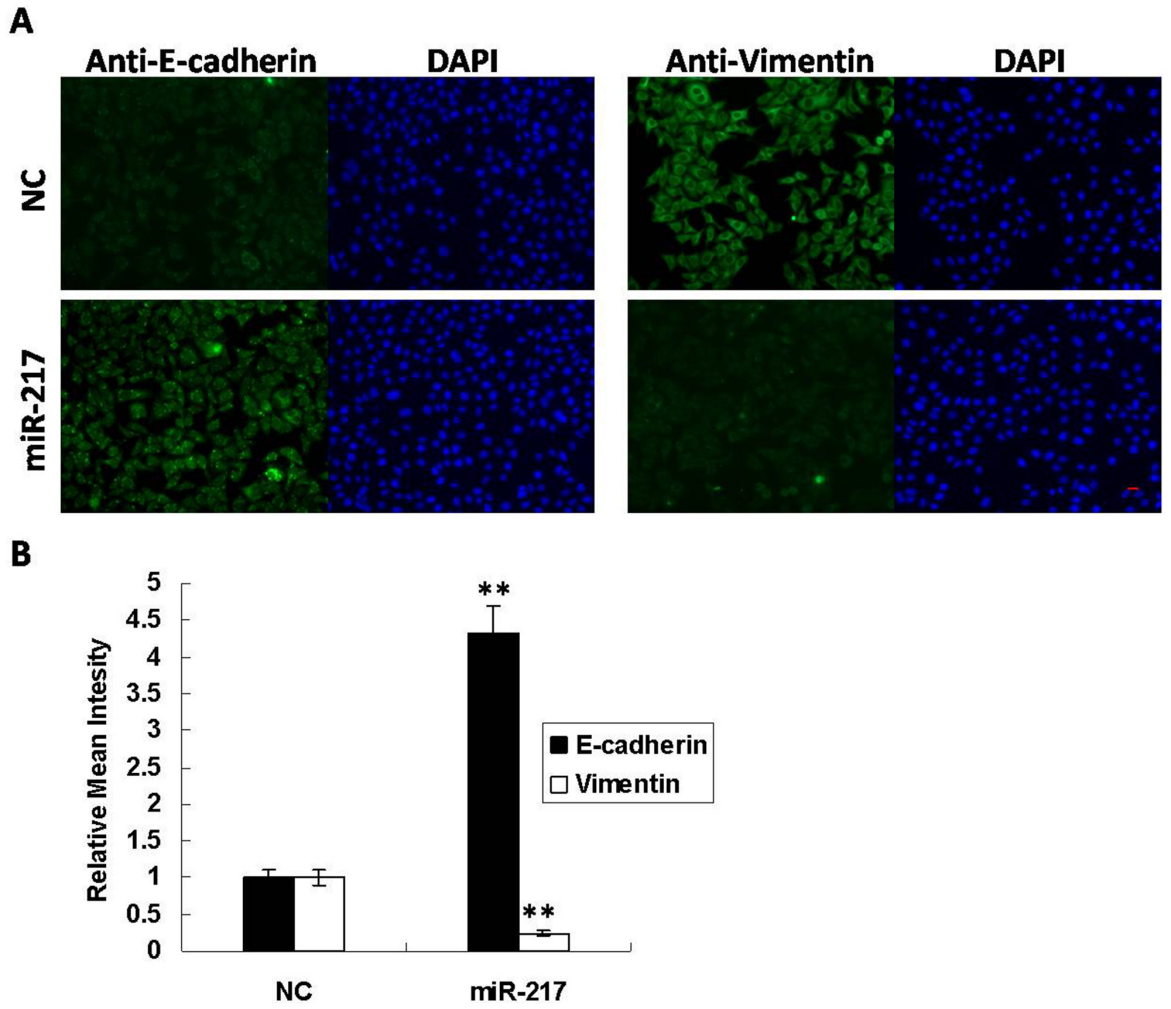
miR-217 inhibits EMT in Hep2 cells **(A)** Overexpression of miR-217 resulted in increased E-cadherin and decreased Vimentin in Hep2 cells. DAPI staining was used to detect nuclei. Scale bar, 50 μm. **(B)** Statistical histogram. N=3.

Epithelial-mesenchymal transition (EMT) is an essential early step in cancer metastasis, and E-cadherin and Vimentin are usually used as EMT markers [15]. Immunofluorescence results demonstrated that E-cadherin was increased by 4-fold while Vimentin was decreased to 30% when miR-217 was overexpressed in Hep2 cells (Figure 3A–3B). And Immunoblotting results represented that overexpression of miR-217 inhibits Snail expression in Hep2 cells (Supplementary Figure 3). These findings revealed that miR-217 functions as a metastatic repressor to suppress EMT.

### miR-217 inhibits tumor angiogenesis in laryngeal cancer cells

**Figure 4:**
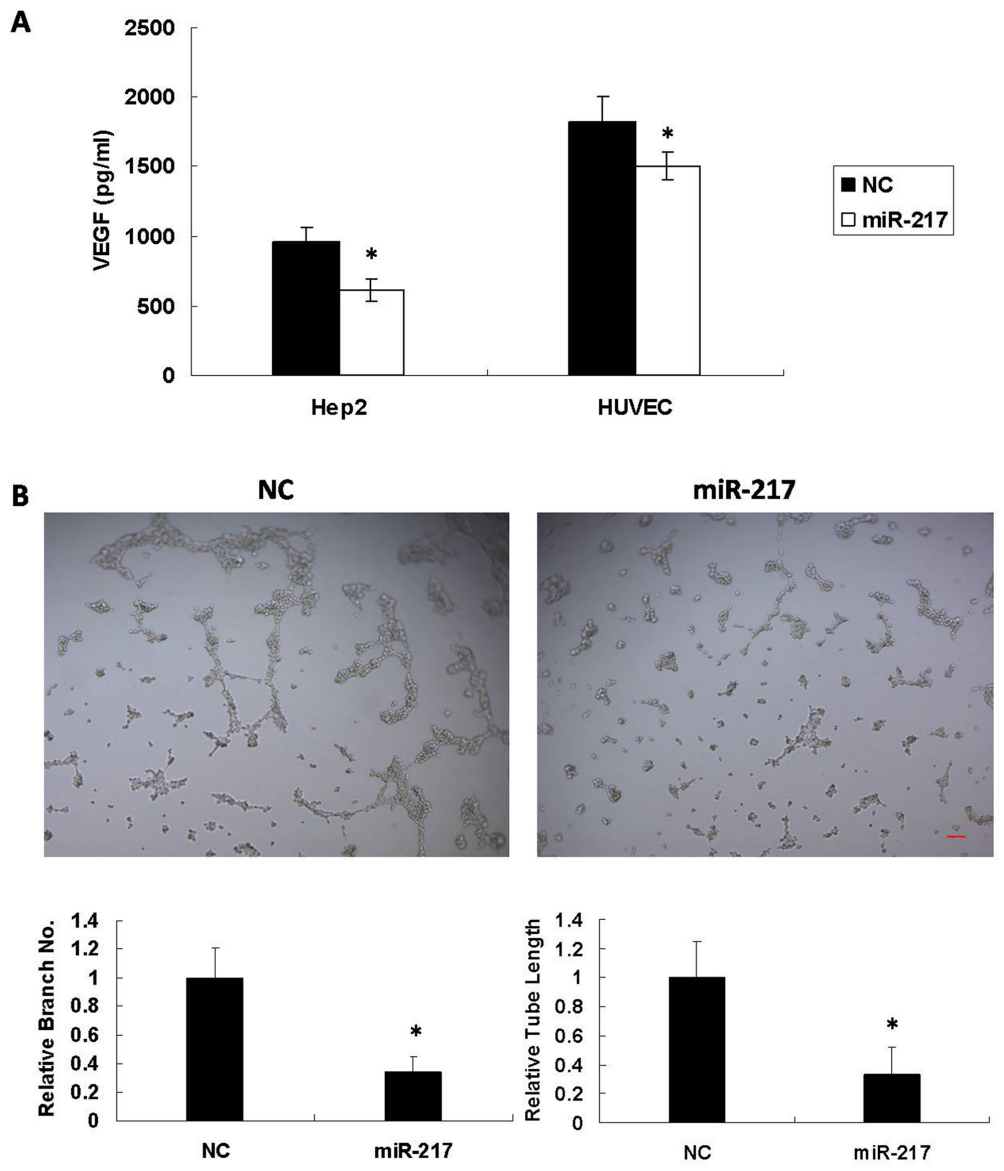
miR-217 inhibits angiogenesis miR-217 inhibits **(A)** VEGF secretion in both Hep2 and HUVEC cells (N=3), and **(B)** tubule elongation and branching formation in HUVEC cells (N=3). Scale bar, 50 μm.

To investigate whether miR-217 functioned as a dominant determinant of angiogenesis, miR-217mimics or NC were transfected into Hep2 and HUVEC cells. 72 h after transfection, the supernatant was collected and analyzed using a VEGF Elisa kit (EH015-96; ExCell Biotech, Suzhou). As shown in Figure 4A, miR-217 reduced VEGF secretion in both Hep2 and HUVEC cells. The transfected HUVEC cells were also seeded on Matrigel as angiogenic stimuli. miR-217 significantly inhibited tubule elongation and branching formation in contrast to NC (Figure 4B).

### miR-217 downregulates its target gene AEG-1 and PD-L1

**Figure 5:**
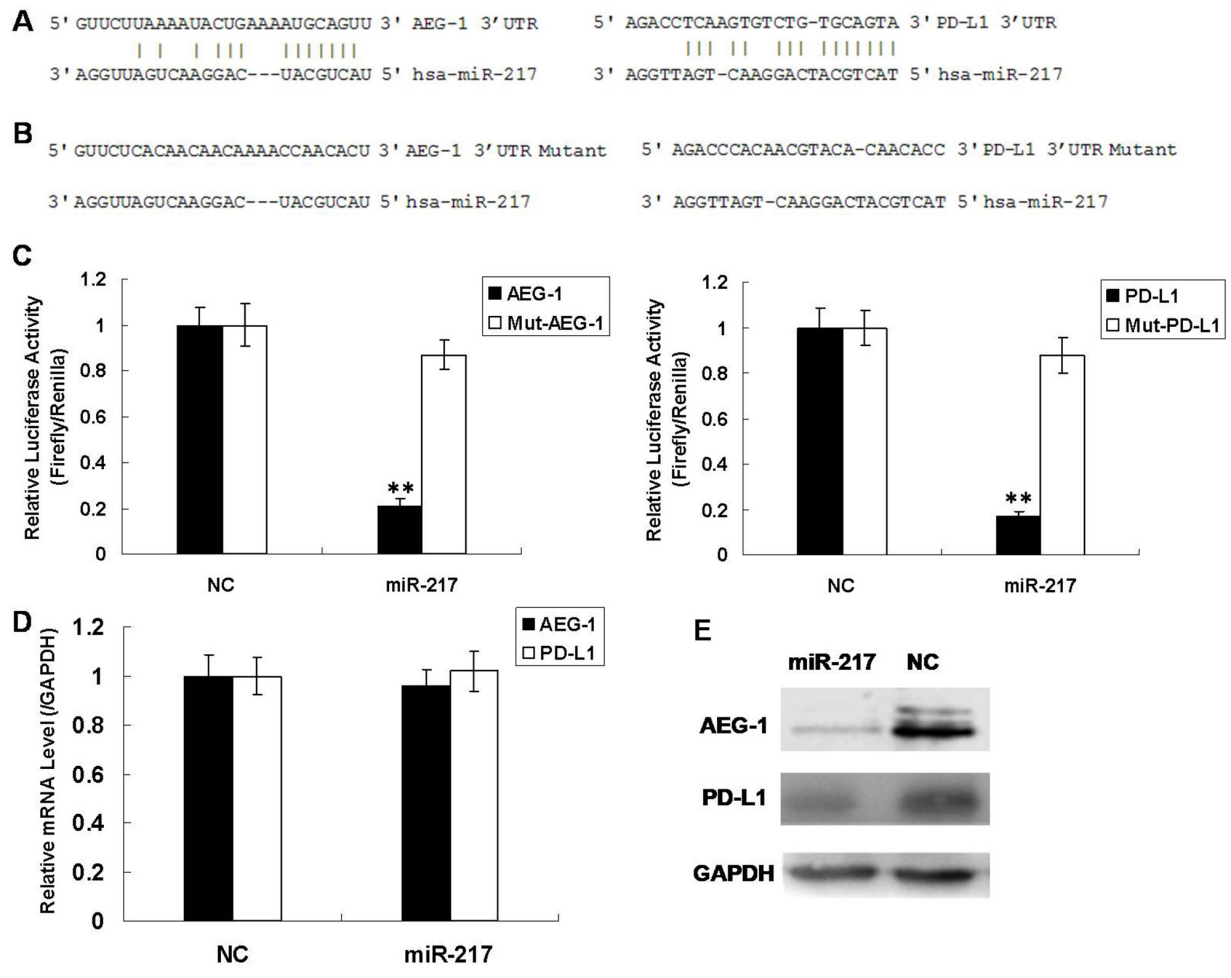
miR-217 directly regulates AEG-1 and PD-L1 in Hep2 cells **(A)** Prediction of miR-217 binding sites on AEG-1 and PD-L1. **(B)** Prediction of miR-217 mutant binding sites on AEG-1 and PD-L1. **(C)** Luciferase activity in Hep2 cells infected with miR-217 or control after transfection with the indicated 3′-UTR-driven reporter plasmid or mutant 3′-UTR-driven reporter plasmid (N=3). **(D)** Real-time PCR results for endogenous AEG-1 and PD-L1 infected with miR-217 or NC (N=3). **(E)** Western blot results for endogenous AEG-1 and PD-L1 infected with miR-217 or NC.

To discover the molecular mechanism of miR-217, we used the miRNA.org website and the RNA22 algorithm to predict its potential target genes [16, 17]. AEG-1 and PD-L1 were predicted to be regulated by miR-217 (Figure 5A). In order to verify whether AEG-1 and PD-L1 were target genes of miR-217, the 3’-untranslated regions (UTR) of AEG-1 and PD-L1 were cloned into a luciferase reporter construct individually (pGL3, Promega). The luciferase activity of AEG-1 decreased by 80% when miR-217 was overexpressed compared to NC and the luciferase activity of PD-L1 was also reduced. (Figure 5C). On the other hand, when the miR-217 binding sites of AEG-1 and PD-L1 were mutated, the luciferase activity was rescued (Figure 5B–5C). Furthermore, we measured the endogenous mRNA and protein levels of AEG-1 and PD-L1 in Hep2 cells when miR-217 was overexpressed, and found that although the mRNA levels of AEG-1 and PD-L1 were unchanged (Figure 5D), the corresponding protein levels were reduced (Figure 5E). This suggests that miR-217 regulates AEG-1 and PD-L1 mainly through repression of translation. In summary, these results confirmed that miR-217 negatively regulates its downstream target genes AEG-1 and PD-L1.

### AEG-1 activates downstream PD-L1

**Figure 6:**
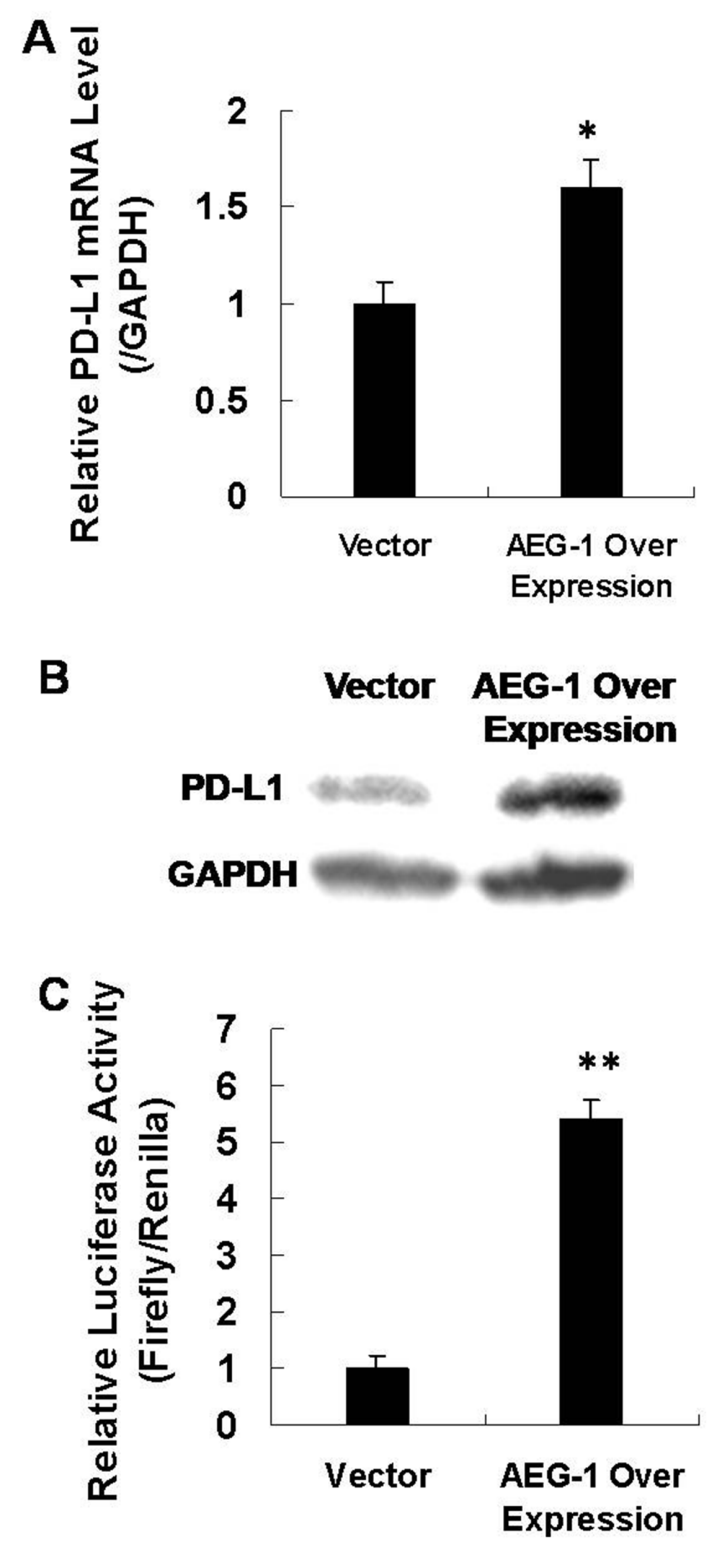
AEG-1 activates downstream PD-L1 **(A)** qRT-PCR assay to measure PD-L1 mRNA levels when AEG-1 was overexpressed (N=3). **(B)** Western blot assay to measure PD-L1 protein levels when AEG-1 was overexpressed . **(C)** Luciferase assay showing activity for co-transfection of AEG-1 and promoter vector in Hep2 cells (N=3).

To investigate the functions of AEG-1 in laryngeal cancer metastasis, we transfected AEG-1 vector into Hep2 cells and measured the PD-L1 mRNA and protein levels using qRT-PCR and western blot assays. We found that when AEG-1 was overexpressed, the levels of PD-L1 mRNA and protein both increased (Figure 6A–6B). Then, we cloned the PD-L1 gene promoter region (3000bp) into a luciferase reporter construct (pGL3-basic, Promega). The luciferase activity of PD-L1 increased 5 fold when AEG-1 was overexpressed compared with controls (Figure 6C). These findings imply that AEG-1 transcriptionally activates PD-L1.

### Expression patterns of AEG-1 and PD-L1 in human laryngeal cancer

**Figure 7:**
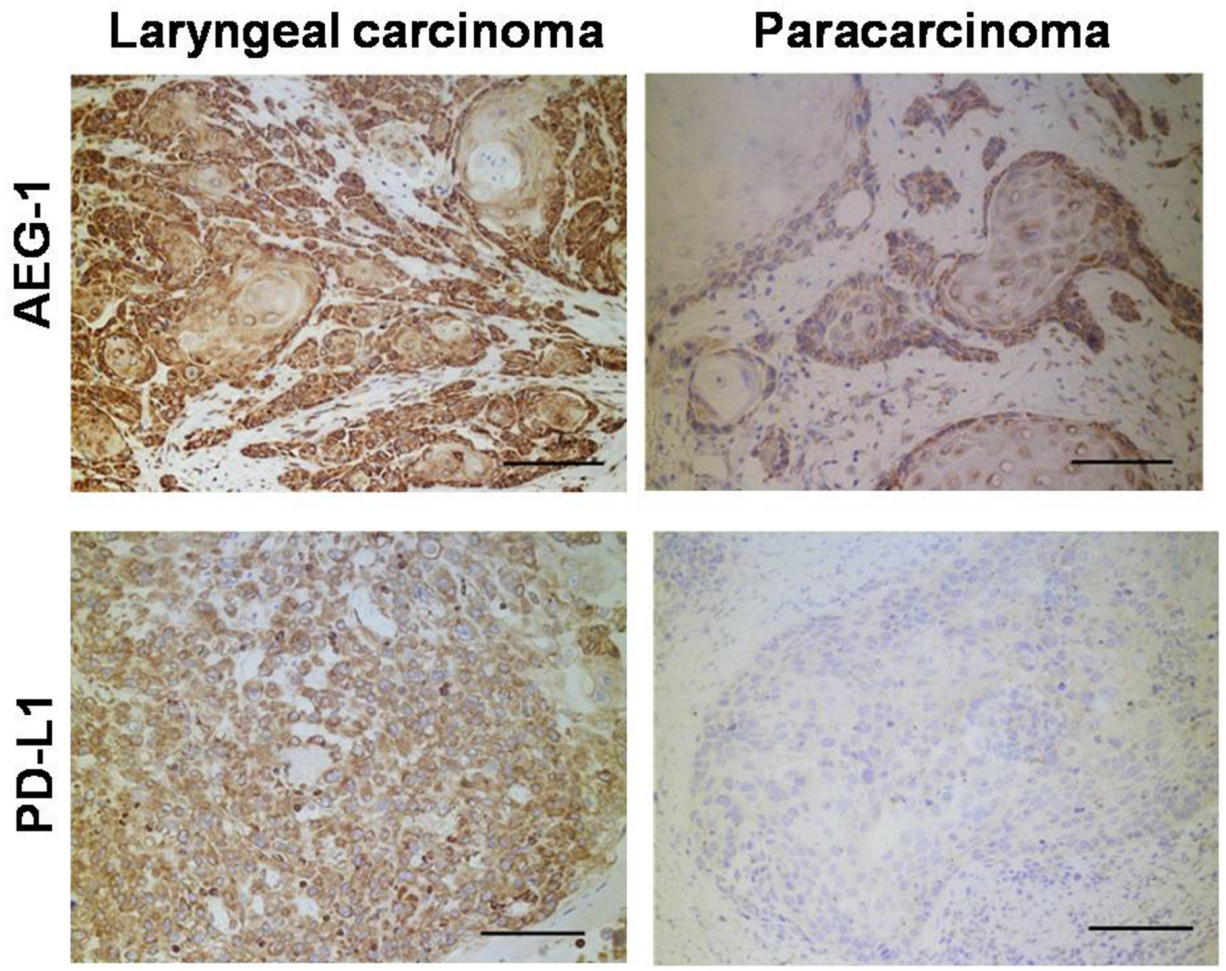
Immunohistochemistry detection of clinical human laryngeal carcinoma samples The number of AEG1 or PD-L1 positive cells was counted in 10 randomly chosen fields for each patient (magnification: ×200). Score: left 3+ and right 1+.

Tissues from patient operations were used to identify gene expression patterns in metastatic laryngeal cancer. As shown in Figure 7, were conducted immunohistochemistry measurements on carcinoma and paracarcinoma tissues. In total, we tested 29 pairs of samples from 29 patients. In all cases, AEG-1 and PD-L1 were expressed at higher levels in carcinoma tissues. Our results revealed that AEG1-positive and PD-L1-positive cells were more frequent in tumor cells than in paracarcinoma cells. (P < 0.01, Mann-Whitney U test). These results suggest that higher levels of AEG-1 and PD-L1 correlate with laryngeal cancer progression, in agreement with findings of our functional experiments.

### miR-217 strongly inhibits laryngeal cancer progression in mouse model

**Figure 8:**
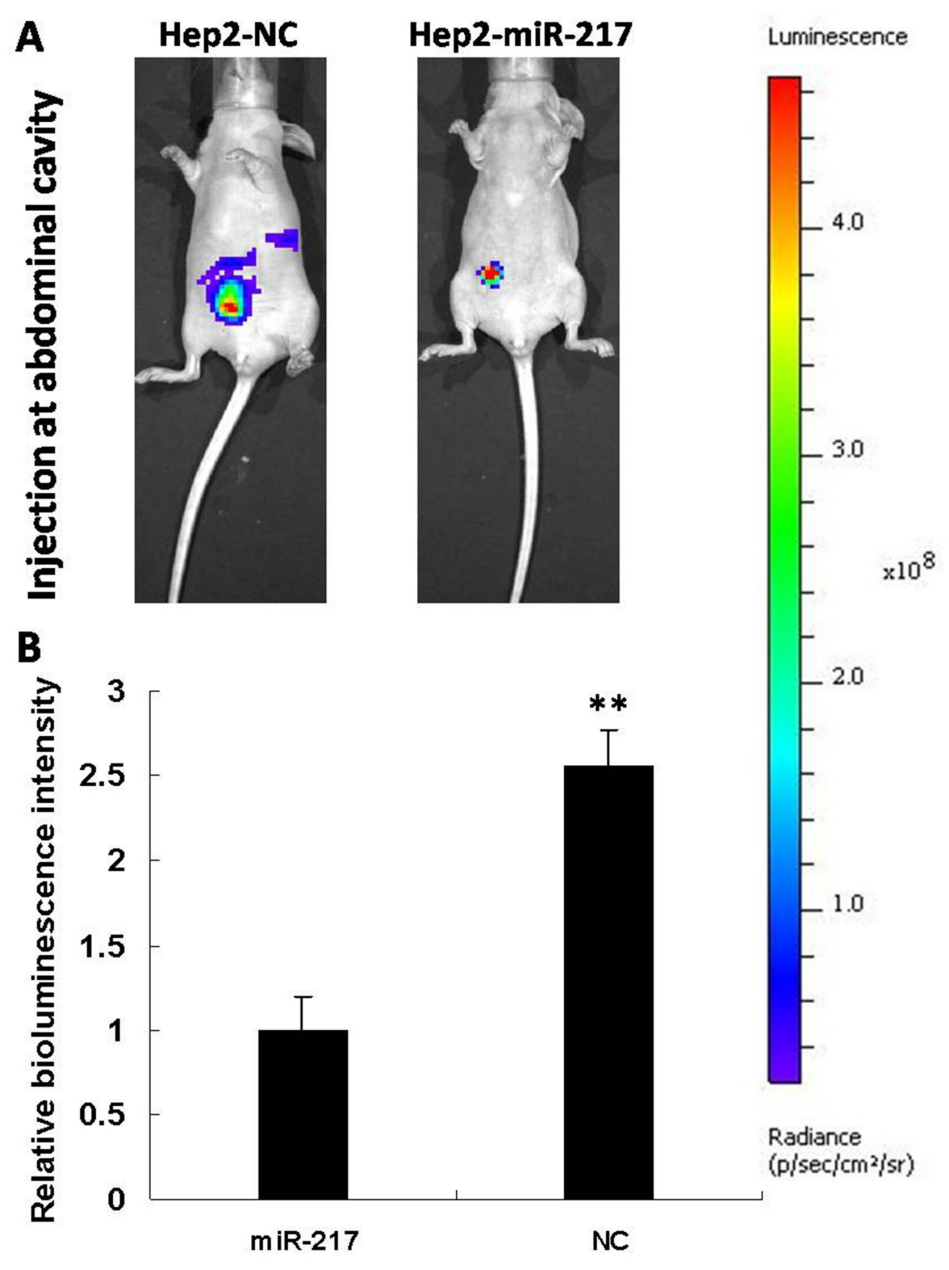
Overexpression of miR-217 strongly inhibits metastasis *in vivo* **(A)** Primary tumor growth upon subcutaneous implantation of 5 × 10^5^ luciferase labeled Hep2 cells infected with the indicated vectors. The experiment was terminated after 6 weeks. **(B)** Statistical histogram (N=10).

In order to further confirm miR-217’s role in laryngeal cancer progression, we constructed a Hep2 cell line stably expressing miR-217 and a control cell line (expressing shRNA with scramble sequence) by transfection with lentivirus. The miR-217 levels in both cell lines were measured by qRT-PCR (Supplementary Figure 4). When cancer cells were injected in the abdominal cavity of nude mice, the miR-217 group (10 mice) showed significantly reduced bioluminescence intensity, compared with the control group (10 mice) (Figure 8). That is, tumor growth and local metastasis capabilities were inhibited by miR-217. These results further confirm that miR-217 is a key player in laryngeal cancer progression.

## DISCUSSION

miRNAs are known to participate in carcinogenesis and metastasis. The essential task is to identify physiologically relevant and therapeutically promising miRNAs [18]. miR-217 has been reported as a potential cancer suppressor in several types of cancers. Zhu *et al* found that miR-217 inhibits cell proliferation and invasion by targeting Runx2 in human glioma [13]. Wang *et al* proved that miR-217 inhibits proliferation, migration, and invasion of esophageal squamous cells by silencing long noncoding RNA MALAT1 [14]. Li *et al* identified miR-217 as a tumor suppressor in human epithelial ovarian cancer by targeting IGF1R [12]. In this study, we investigated the role of miR-217 in laryngeal carcinoma. Strikingly, miR-217 levels in laryngeal carcinoma tissues were significantly decreased compared to those in paired normal tissues. Then, our functional studies revealed that miR-217 inhibited cell migration, invasion and proliferation, and induced cell apoptosis and G1 phase arrest in Hep2 cells. Furthermore, we demonstrated that miR-217 inhibited EMT and angiogenesis.

In view of the powerful inhibitory effect on carcinogenesis that miR-217 has in Hep2 cells, we used a bioinformatics approach to identify potential target genes. We then confirmed AEG-1 and PD-L1 as target genes of miR-217 by use of luciferase assay, qRT-PCR assay and western blot. Human AEG-1 is a protein with 582 amino acids and a molecular mass of 64 kDa that activates the downstream PI3k/Akt, NF-kB and Wnt pathways [19]. AEG-1 is involved in metastasis for most types of cancer, including colorectal cancer, ovarian cancer, and breast cancer [20–22]. We also investigated effects of AEG-1 on metastasic traits, including cell migration, cell invasion, cell cycle and angiogenesis, through RNAi tools. We found that silencing of AEG-1 resulted in reduced migratory and invasive capability as well as reduced angiogenesis and cell cycle arrest (Supplementary Figures 5 and 6). So we can draw a deduction that miR-217 inhibits metastasic traits, including cell migration, cell invasion, cell cycle and angiogenesis, by repressing target gene AEG-1.

Li *et al* discovered that AEG-1 acts downstream of AKR1C2 and NF1 in liver cancer [23]. In this study, we discovered that PD-L1 is target gene for miR-217 as well as a downstream target of AEG-1. AEG-1 promotes PD-L1 activation at the transcriptional level. Targeting immune checkpoints such as programmed cell death protein 1 (PD1) and programmed cell death 1 ligand 1 (PD-L1) yields notable benefits in the treatment of most types of cancer by blocking immune-inhibitory signaling and enabling patients to produce an effective anti-cancer immune-response [24]. Recently, Vassilakopoulou *et al* found that PD-L1 levels correlate with better outcome in laryngeal cancer [25]. Here, our results suggest the existence of an miR-217/AEG-1/PD-L1 signaling pathway in laryngeal cancer. Finally, our clinical sample immunohistochemistry data and the results from our mouse model experiments further substantiated our findings described above.

In conclusion, our findings contribute to understanding the role that dysregulated miRNAs play in human laryngeal cancer progression, especially in metastasis. Our results provide insight into the physiological significance of miRNAs in human laryngeal carcinoma, thereby opening novel therapeutic avenues.

## MATERIALS AND METHODS

### Cell culture and transfection

We obtained Hep2 cells from the American Type Culture Collection (ATCC, Manassas, VA, USA) and cultured them in Dulbecco’s Modified Eagle’s Medium containing 10 % fetal bovine serum, 100 U ml^−1^ penicillin and 0.1 mg ml^−1^ streptomycin under humidified conditions of 95 % air and 5 % CO_2_ at 37 °C. The transfection protocol was followed with the Lipofectamine™ 3000 (Invitrogen) transfection reagent instructions.

### RNAi and plasmid

miRNA mimics were obtained from the View-Solid Biotech (Beijing). During RNAi experiments, we used a scrambled siRNA as a negative control (NC). The human AEG-1 cDNA clone was constructed into the pcDNA3.1 vector (Addgene). During overexpression experiments, we used the empty vector as a negative control (Vector).

### Transwell migration and invasion assay

For migration assays, Hep2 cells were seeded into the upper chamber of a transwell insert (pore size, 8 µm; Costar) in 100 μl of serum-free medium per well. Medium at a volume of 600 μl, containing 10 % serum, was placed in the lower chamber to act as a chemoattractant. Non-migratory cells were removed from the upper chamber by scraping with a cotton bud. The cells remaining on the lower surface of the insert were fixed with 4 % formaldehyde (Sigma) and were stained by DAPI (Roche). For invasion assays, cells were seeded in a Matrigel (Bio-Rad)-coated chamber and were incubated at 37 °C.

### Cell proliferation assay

Hep2 cells were seeded in a 96-well plate at a density of 3000 cells per well. After one day, cells were transfected with the indicated miRNAs or siRNAs. Two days later, cells were incubated with 10 % CCK8 reagent (DoJinDo Laboratories, Japan) for 1 h at 37 °C. Then, plates were analyzed using the automatic spectrometer (Multimode Reader; Enspire) at 450nm.

### Cell apoptosis assay

Apoptosis was measured by propidium iodide and annexin V–FITC staining, which was performed as described by the manufacturer (Roche Inc.). Briefly, 1×10^6^ Hep2 cells were washed in PBS and resuspended in 100 ul of incubation buffer which was added with 2 ul annexin V-FITC labeling reagent and 2ul propidium iodide solution. The mixture was incubated for 10 min. Then the cells were analyzed on a flow cytometer.

### Cell cycle assay

1×10^6^ Hep2 cells were washed in PBS and resuspended in 500 ul of 70% alcohol for 2 hours at 4 °C. Then the cells were washed in PBS and resuspended in 200 ul propidium iodide staining solution. The mixture was incubated for 20 min. Then the cells were analyzed on a flow cytometer.

### HUVEC tube formation assay

HUVEC cells were seeded in a 96-well Matrigel-coated plate at a density of 3×10^4^ cells per well and incubated for 8 h at 37 °C. The cells were then analyzed using a light microscope.

### Immunofluorescence

Hep2 cells were seeded onto sterile cover slides and allowed to attach overnight. The cells were then fixed with 4 % formaldehyde, permeabilized with 0.1 % Triton X-100 and blocked in 2 % bovine serum albumin for 1 h at room temperature. The expression of E-cadherin and vimentin was examined using targeted antibodies (anti-E-cadherin: ab76055; Abcam) (anti-vimentin: ab8978; Abcam) and visualized using anti-rabbit IgG (H + L), F (ab)2 fragment (Alexa Fluor 488 Conjugate, Cell Signaling Technology). The antibodies were diluted for 1:200. The cell nucleus was stained with DAPI (Roche). Immunofluorescence was assessed using a light microscope (EcliPSE Ti-U; Nikon).

### Luciferase assay

For the luciferase assay, 4.0 × 10^4^ Hep2 cells were co-transfected with 200 ng of miRNA mimics, 200 ng of the indicated pGL3 firefly luciferase construct and 20 ng of a pGL3 renilla luciferase construct as normalization control. The medium was changed 6 h post-transfection and luciferase activity was measured after 48 h using the dual luciferase reporter assay system (Promega).

### Immunoblotting

Lysates were resolved by electrophoresis, transferred to a poly-vinylidene difluoride membrane (Millipore Corporation), and probed with antibodies against AEG-1 (ab45338; Abcam) or PD-L1 (ab205921; Abcam). The antibodies were diluted for 1:1000.

### Human laryngeal sample and immunohistochemistry

All human laryngeal samples were obtained from the Tumor Hospital affiliated Harbin Medical University. Before surgery at the center, all patients provided written informed consent to allow for any excess tissue to be used for research studies. Immunohistochemistry (IHC) was performed at the Tumor Hospital affiliated Harbin Medical University for IHC, formalin-fixed, paraffin-embedded sections (4 µm thick) of tissue, were blocked with 1% H2O2 and then subjected to antigen retrieval in trypsin for 30 min at 37 °C; followed by immersion in EDTA buffer (pH 9.0, Maixin, China) for 20 min at 120 °C in an autoclave. Sections were then blocked with Protein Blocking Agent (Streptavidin-Biotin Universal Detection System, Beckman Coulter, Marseille, France) and incubated with the following primary antibodies overnight at 4 °C: rabbit anti-human AEG1 (13860-1-AP, 1:100, IgG, Proteintech group, USA) or rabbit anti-human PD-L1 (17952-1-AP, 1:100, IgG, Proteintech group, USA). This was followed by incubation with secondary antibodies from the Streptavidin-Biotin Universal Detection System (Beckman Coulter). Sections were visualized using DAB. Specific isotype control antibodies and phosphate-buffered saline (PBS; omitting primary antibodies) were used as negative controls. The number of cells staining positive by IHC were scored as 0 (absent), 1+ (< 25% of cells), 2+ (25–50% of cells), 3+ (50–75% of cells), or 4+ (> 75% of cells). The scoring was done in a blinded fashion.

### RNA extraction and quantitative real-time PCR

For clinical samples and cultured cell lines, total RNA was purified by using the Trizol kit (Tiangen Biotech, Beijing) according to the manufacturer’s protocols. Primers for reverse transcription and PCR were from hsa-miR-217 detection kit (Ribo Biotech, Guangzhou). miR-217 levels were quantified by qRT-PCR with the SYBR Premix Ex Taq Kit (Takara). Primers were listed in Supplementary Table 1. qRT-PCR was performed in a DNA Engine Opticon2 system (Bio-Rad). The following PCR protocol was used: denaturation at 95 °C for 3 min, amplification for 40 cycles of 95 °C for 12 s and 62 °C for 40 s. The melting curve was plotted from 62 °C to 95 °C, read every 0.2 °C with a 2 s hold. U6 small nuclear RNA was used as an internal control. The results were represented as fold changes, which were calculated by the 2^-ΔCT^ method.

### Animal study

All research involving animals complied with the protocols approved by the Laboratory Animal Center, Peking University. All BALB/C nude mice were purchased from the animal center of Peking University. For tumor implantation, luciferase and GFP labeled Hep2 cells were suspended in PBS and injected at abdominal cavity of mice (5*10^5^ cells per mouse). After 6 weeks, metastasis status was examined by In-Vivo Imaging System (IVIS Spectrum CT, Caliper Life Sciences) with the substrate Stop Glo Buffer (E641A, Promega).

### Statistical analysis

All of the results are expressed as the means and totals derived from independent experiments. When comparing two groups, Student’s unpaired t-test (two-tailed) was used. For all of the tests, a P value <0.05 was considered significant. The Benjamini and Hochberg false discovery rate was used as a correction for multiple testing. * indicates P<0.05; ** indicates P<0.01. Error bars represent the SDs of at least three independent experiments.

## SUPPLEMENTARY MATERIALS FIGURES AND TABLES


