# Supplementary material for: miR-217 inhibits laryngeal cancer metastasis by repressing AEG-1 and PD-L1 expression
Source: Oncotarget. 2017 Jul 10;8(37):62143–53. doi: 10.18632/oncotarget.19121 (PMC5617493; doi:10.18632/oncotarget.19121)
Supplement: Supplementary file 1 [file oncotarget-08-62143-s001.pdf]

miR-217 Inhibits Laryngeal Cancer Metastasis by Repressing AEG-1 and PD-L1 Expression

SUPPLEMENTARY MATERIALS

Caspase-3 activity assay

Hep2 cells were seeded in a 6-well plate at a density of 5×10^5 cells per well. After one day, cells were transfected with the indicated miRNAs or siRNAs. Two days later, lysates were incubated with Caspase-3 activity assay reagent (Beyotime Biotech, China). Then, plates were analyzed using the automatic spectrometer (Multimode Reader; Enspire) at 405nm.

Immunoblotting

Lysates were resolved by electrophoresis, transferred to a poly-vinylidene difluoride membrane (Millipore Corporation), and probed with antibodies against Snail (13099-1-AP; Proteintech). The antibodies were diluted for 1:500.

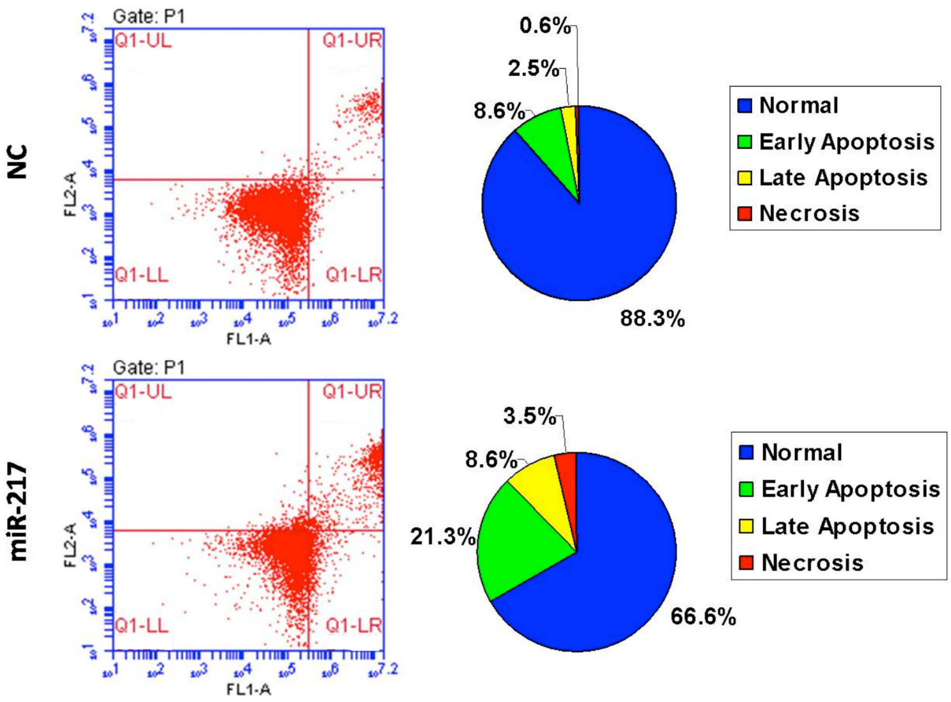

Supplementary Figure 1: Annexin V/PI staining assay showing that overexpression of miR-217 promotes apoptosis and necrosis in Hep2 cells.

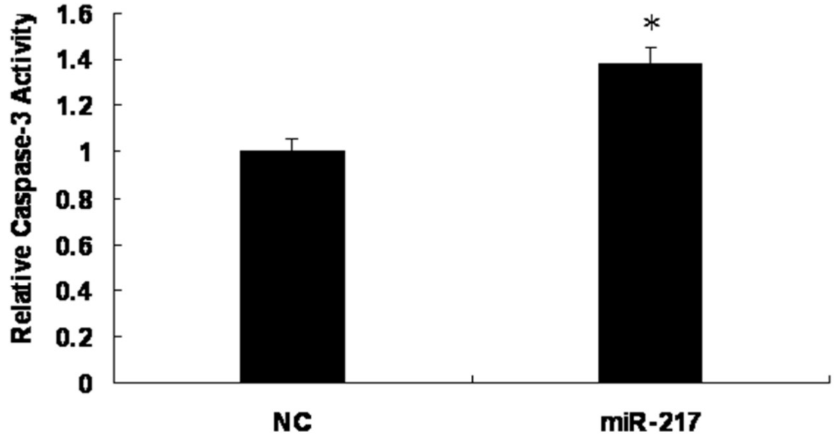

Supplementary Figure 2: Caspase-3 activity assay showing that overexpression of miR-217 promotes cell apoptotic activity in Hep2 cells.

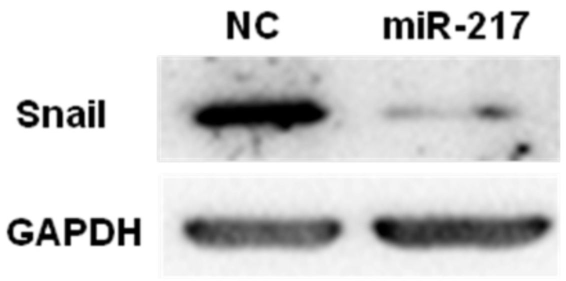

Supplementary Figure 3: Immunoblotting results showing that overexpression of miR-217 inhibits Snail expression in Hep2 cells.

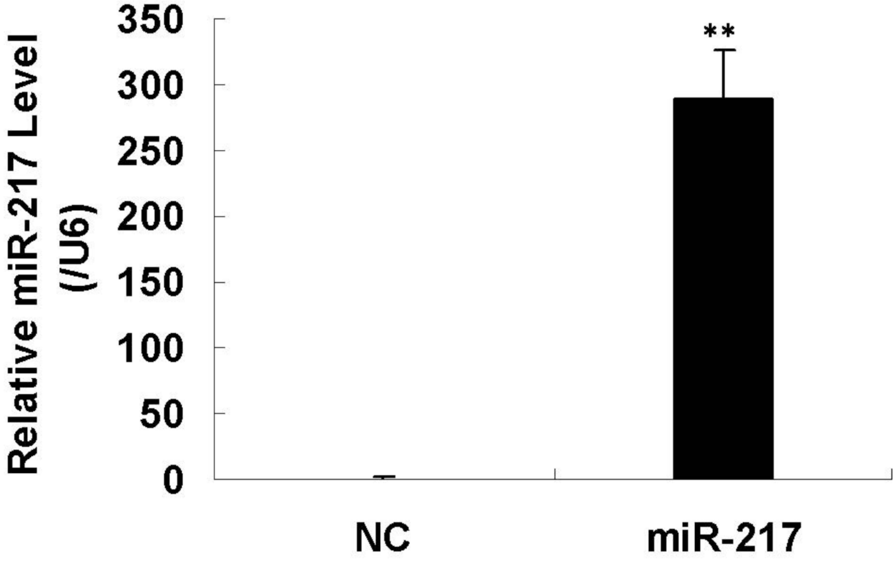

Supplementary Figure 4: Detection of miR-217 stably expressed in Hep2 cells. qRT-PCR results showing that miR-217 levels can be efficiently elevated in our Hep2 cell line. N=3.

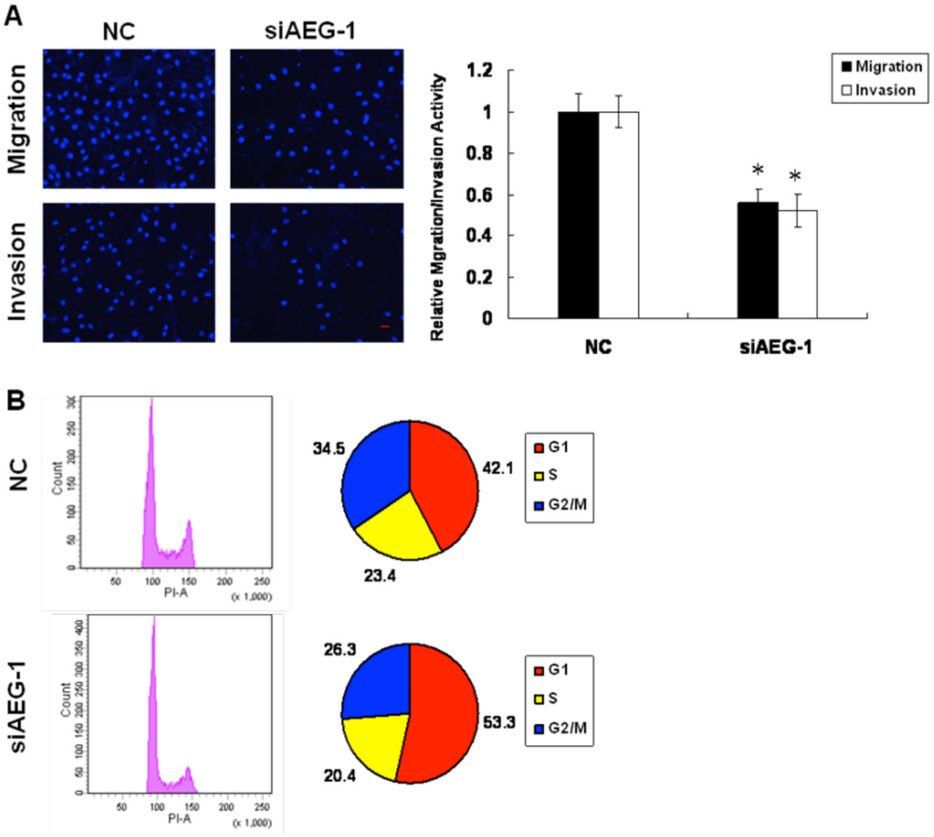

Supplementary Figure 5: AEG-1 modulates metastatic traits in Hep2 cells. Silencing of AEG-1 in Hep2 cells resulted in **(A)** reduced migratory and invasive capability (N=5; scale bar, 50 μm), and **(B)** cell cycle arrest (N=3).

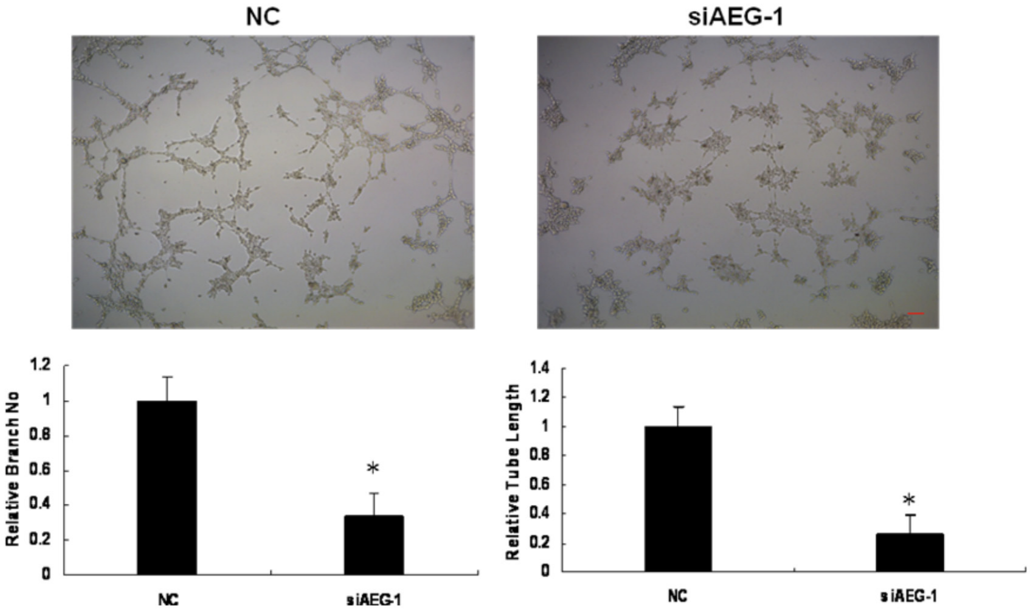

Supplementary Figure 6: AEG-1 modulates angiogenesis. Silencing of AEG-1 inhibits tubule elongation and branching formation in HUVEC cells (N=3). Scale bar, 50 μm.

Supplementary Table 1: Primers for real-time PCR assays.

Gene	Primer	Sequence
AEG-1	AEG1-F	CCTGGCCTTGCTGAAGAATC
	AEG1-R	GGCTGCTTTGCTGTTACACT
PD-L1	PD-L1-F	TATGGTGGTGCCGACTACAA
	PD-L1-R	TGCTTGTCCAGATGACTTCG
GADPH	GADPH-F	CATGAGAAGTATGACAACAGCCT
	GADPH-R	AGTCCTTCCACGATACCAAAGT
